# Nature-Based Solutions in Workplace Settings: A Scoping Review on Pathways for Integrated Quality, Environmental, Health, and Safety Management

**DOI:** 10.3390/ijerph22091455

**Published:** 2025-09-19

**Authors:** Marcos Vinícius de Castro, Rogerio Galante Negri, Fabiana Alves Fiore, Adriano Bressane

**Affiliations:** 1 Graduate Program in Civil and Environmental Engineering, São Paulo State University, Eng. Luís Coube Avenue, 2085, Bauru City 17033-360, Brazil; 2 Institute of Science and Technology, São Paulo State University, Presidente Dutra Highway, Km 137,8, São José dos Campos City 12247-004, Brazil; rogerio.negri@unesp.br (R.G.N.); fabiana.fiore@unesp.br (F.A.F.)

**Keywords:** nature-based solutions, occupational health, workplace setting, biophilic design, environmental quality

## Abstract

Occupational environments often expose workers to physical and psychological stressors that compromise well-being and productivity. While biophilic design has gained attention, there remains limited systematic integration of Nature-Based Solutions (NbS) within workplace management frameworks. This review aims to map the empirical impacts of NbSs on occupational health, productivity, and environmental quality, and to identify key barriers and facilitators for their integration into comprehensive Quality, Environmental, Health, and Safety (QEHS) management systems. A scoping literature review was conducted in accordance with the PRISMA-ScR (Preferred Reporting Items for Systematic reviews and Meta-Analyses extension for Scoping Reviews) guidelines. A comprehensive search was performed in the Scopus and Web of Science databases for studies published between 2019 and 2024. A total of 2452 records were initially retrieved, with 39 studies retained for synthesis following screening, eligibility assessment, and critical appraisal using the Joanna Briggs Institute checklist. Findings indicate that NbSs can reduce stress, improve physical and cognitive health, and enhance workplace productivity. Reported benefits include reduced absenteeism, improved indoor air quality, and measurable financial returns. However, significant challenges persist, including high upfront costs, ongoing maintenance demands, a shortage of specialized labor, and methodological heterogeneity across studies. In particular, hybrid approaches combining physical natural elements and immersive technologies such as virtual reality emerged as promising alternatives for spatially constrained environments. Participatory co-design and stakeholder engagement were also identified as critical success factors for effective implementation. Integrating NbSs into QEHS frameworks has the potential to foster healthier, more resilient, and sustainable workplaces. Alignment with recognized certifications can further support systematic adoption and monitoring. Future research should prioritize longitudinal designs, standardized outcome metrics, and physiological markers, while addressing geographical gaps through studies in underrepresented regions. Embedding participatory processes and certification alignment can enhance stakeholder buy-in and practical scalability, advancing the integration of NbSs into holistic workplace management strategies.

## 1. Introduction

Nature-Based Solutions (NbS) are increasingly recognized as innovative, sustainable strategies capable of addressing multifaceted environmental, social, and economic challenges [1,2]. Grounded in ecological principles, NbSs integrate natural processes and materials, such as vegetation, natural lighting, and organic components, into built environments to promote biodiversity, resilience, and human well-being [3]. While their transformative potential in urban design and ecosystem restoration is well-documented, their application in workplace settings remains limited, despite growing evidence of benefits for human health, cognitive performance, and environmental quality [4].

The absence of nature in occupational environments, as highlighted by Gillis and Gatersleben [5], compromises occupant health and well-being. Integrating natural elements, such as indoor plants and natural materials, creates healthier spaces, enhancing comfort and resilience. Recent studies confirm that biophilic design enhances psychological well-being, reduces stress, and supports cognitive function [6,7,8]. Notably, Bratman et al. [9] reported that exposure to nature is associated with reductions in stress, as measured by self-report and physiological biomarkers, and with improvements in mood and psychological well-being.

This is especially relevant given the increasing complexity of occupational environments, where poor air quality, noise, and inadequate lighting persist as common stressors [10]. Integrating NbSs within Quality, Environmental, Health, and Safety (QEHS) management systems can enhance worker well-being, reduce absenteeism, and improve organizational outcomes. Additionally, Alaghbari and Beshr [11] emphasize that adopting green management practices enhances organizational sustainability by integrating environmental, social, and economic dimensions. Their findings highlight the role of CSR, green innovation, and management commitment in fostering resilience at the organizational level.

Despite promising findings, empirical research on NbSs in occupational contexts remains limited. Existing literature focuses primarily on public and residential spaces, underutilizing the potential of NbSs in workplace environments. Nonetheless, a growing body of evidence supports the integration of biophilic principles to address workplace challenges such as air quality, thermal comfort, and psychological health [6].

From a scientific perspective, investigating NbSs in occupational settings addresses a critical knowledge gap. Given that adults spend the majority of their waking hours at work, leveraging NbSs could substantially enhance well-being and organizational efficiency [6,12]. Petrova et al. [4] further note that NbSs can sustainably improve environmental quality and ecosystem services, offering long-term strategies that promote employee life quality.

For both industry and society, implementing NbSs presents dual advantages: improving employee health and advancing sustainability goals. Green workplaces enhance employee well-being and can align with corporate social responsibility and sustainability objectives [13]. As Collier and Bourke [14] argue, integrating NbSs into planning and ongoing management fosters more sustainable and resilient environmental systems, with potential long-term social co-benefits.

This study seeks to bridge existing gaps by proposing a comprehensive framework for NbS integration into QEHS strategies. By evaluating the interaction between natural elements and workplace dynamics, it aims to generate actionable insights to inform practice, shape policy, and support healthier, more sustainable, and productive work environments [15]. The research is guided by two questions, which have been structured according to the PICO (Population, Intervention, Comparison, Outcome) framework: (1) Among occupational populations, what is the impact of implementing NbSs on indicators of physical and mental health, productivity, and environmental quality? (2) In organizations operating under integrated QEHS management systems, what barriers and facilitators affect the successful implementation of NbSs?

To ensure rigor and transparency, this study adopts a scoping literature review (SLR), structured in accordance with the PRISMA-ScR (Preferred Reporting Items for Systematic reviews and Meta-Analyses extension for Scoping Reviews) checklist [16]. This approach consolidates and maps existing evidence on NbS applications in occupational settings, thereby advancing theoretical understanding and informing practical implementation frameworks [12].

It is worth noting that in this study, NbS refers to a broad range of interventions grounded in ecological principles that integrate natural processes and elements into built environments, with the aim of generating co-benefits for human well-being and environmental quality [3]. In turn, biophilic design, as discussed by Gillis and Gatersleben [5], focuses on architectural and design strategies that incorporate natural elements, such as vegetation, natural materials, light, and water, into interior environments to support occupant well-being. More recently, it has been framed in the literature as a complementary approach within the broader scope of NbSs. The term nature exposure encompasses both passive and active interactions with natural environments, whether physical or virtual, and is conceptualized here as an experiential outcome of NbSs and biophilic interventions.

## 2. Background

NbSs are grounded in ecological principles, emphasizing co-benefits for humans and the environment. Fundamentally, these solutions are characterized by their focus on sustainability, aiming to ensure long-term ecological resilience while minimizing environmental degradation [17]. They simultaneously deliver co-benefits, generating positive outcomes for both human well-being and biodiversity. Furthermore, they exhibit adaptability, enabling interventions to be tailored to specific local contexts and to remain flexible under changing environmental and social conditions [18]. Ecosystem-based management is also central, advocating holistic approaches that sustain ecosystem services over time.

NbSs have been successfully implemented across a wide range of sectors, demonstrating notable versatility and effectiveness. Green roofs and walls, for example, play a significant role in mitigating urban heat islands and improving air quality, while also providing indirect cultural and wellbeing benefits [19,20]. Urban wetlands, as implemented in cities like Singapore and Melbourne, offer efficient stormwater management, reduce flood risks, and enhance recreational spaces for urban residents [21]. In riparian buffers composed of vegetation along waterways effectively reduce nutrient runoff and protect aquatic ecosystems from degradation [21].

Coastal areas have benefited extensively from mangrove restoration projects, particularly in Southeast Asia, where these ecosystems function as natural barriers against storm surges while providing crucial habitats for marine biodiversity. Coral reef restoration initiatives in the Caribbean and Pacific regions have demonstrated the potential to support local fisheries, promote tourism, and strengthen coastal defenses [22].

The adaptability of NbSs to diverse environmental, social, and economic contexts underscores their transformative potential. However, their application in occupational environments remain largely underutilized, representing a promising frontier for innovation and sustainability in workplace design and management.

### 2.1. Occupational Environments and Challenges

Occupational environments significantly influence workers’ physical health, mental well-being, and overall productivity. Poorly designed workplaces often expose employees to stressors such as inadequate lighting, poor air quality, excessive noise, and thermal discomfort. These environmental factors are closely linked to adverse health outcomes, including elevated stress, fatigue, and respiratory illnesses, ultimately undermining workplace productivity [23].

Emerging research highlights the critical relationship between workplace environments and employee performance. For instance, natural light has been shown to regulate circadian rhythms, thereby improving sleep patterns and cognitive performance [24]. Additionally, the integration of greenery within workspaces significantly reduces stress and enhances creativity and cognitive function [6].

Although the benefits of integrating nature-based elements into workplaces are increasingly documented, many office environments, particularly in urban and industrial contexts, remain driven by operational and energy efficiency targets, often overlooking the environmental conditions essential to occupant health and well-being [25]. Compounding this issue, the global rise in sedentary work patterns has been directly associated with heightened risks of physical inactivity, musculoskeletal disorders, and mental health challenges [26].

In response to these multifaceted challenges, organizations have adopted a variety of strategies aimed at fostering healthier and more sustainable occupational environments. Biophilic design, for instance, involves the deliberate incorporation of natural elements such as plants, natural materials, and daylight into architectural spaces, a practice shown to improve emotional well-being, stimulate creativity, and reduce absenteeism [27]. Efforts to enhance indoor air quality through advanced HVAC systems have also been pursued, although these technological solutions often fail to capture the full ecosystemic benefits offered by NbSs, which can simultaneously purify air and elevate psychological comfort [12].

Moreover, the adoption of green certification frameworks, such as LEED and WELL, has encouraged more sustainable building practices and healthier indoor environments. Nonetheless, these certifications frequently fall short in integrating the multifunctional potential of NbSs into their criteria [28]. Mental health programs, including mindfulness training and flexible work arrangements, have become increasingly prevalent. However, current initiatives show mixed evidence and methodological limitations, which restrict a full understanding of the restorative potential of nature-connected spaces [13].

Despite growing awareness of these strategies, organizations continue to face significant barriers, notably cost constraints, limited knowledge, and insufficient access to specialized expertise. These limitations underscore the considerable untapped potential of NbSs as scalable and integrative solutions capable of concurrently enhancing occupational health, productivity, and environmental quality [23].

### 2.2. Existing Evidence on NbS Benefits

Although there is substantial evidence supporting the benefits of NbSs in non-occupational contexts, their integration into workplace environments remains insufficiently explored. This oversight is particularly notable, given that many of the challenges NbSs effectively address, such as inadequate air quality, thermal discomfort, and psychological strain, are also prevalent in work settings [12]. Preliminary studies, however, suggest that NbSs could be transformative for occupational health and organizational performance.

In terms of physical health, the integration of greenery and natural light within office spaces has been associated with improvements in sleep quality, both of which contribute to overall physical well-being [6]. Regarding mental well-being, exposure to natural elements in the workplace has been associated with reduced stress and improved self-reported well-being, though evidence remains mixed and largely short-term [13].

Environmental quality also benefits from the adoption of NbSs, as the incorporation of indoor green walls and natural ventilation systems enhances air circulation and attenuates ambient noise levels, thereby creating more comfortable and cognitively supportive workspaces [12,27]. Furthermore, at the organizational level, NbSs have the potential to enhance workplace well-being and employee satisfaction, and they may support broader sustainability goals [13].

While these preliminary findings are promising, there remains a clear gap in systematic research regarding the implementation and outcomes of NbSs in occupational settings. Addressing this gap is essential to establish empirical foundations and develop practical frameworks that enable organizations to successfully integrate NbSs into workplace design and management practices.

## 3. Methodology

This study employed a SLR to examine the multifaceted benefits associated with the implementation of NbSs, with particular emphasis on occupational health, psychological well-being, organizational productivity, and environmental sustainability. The scoping review was conducted in accordance with the Preferred Reporting Items for Systematic reviews and Meta-Analyses extension for Scoping Reviews (PRISMA-ScR) checklist [16] to ensure methodological rigor and transparency. The Scopus and Web of Science databases were selected for their comprehensive multidisciplinary coverage and extensive indexing of peer-reviewed studies. A strategic combination of search terms, including ‘Nature-Based Solutions’ and ‘Nature Exposure’, was applied using Boolean operators to maximize the relevance and breadth of retrieved records (Table 1).

**Figure 1:**
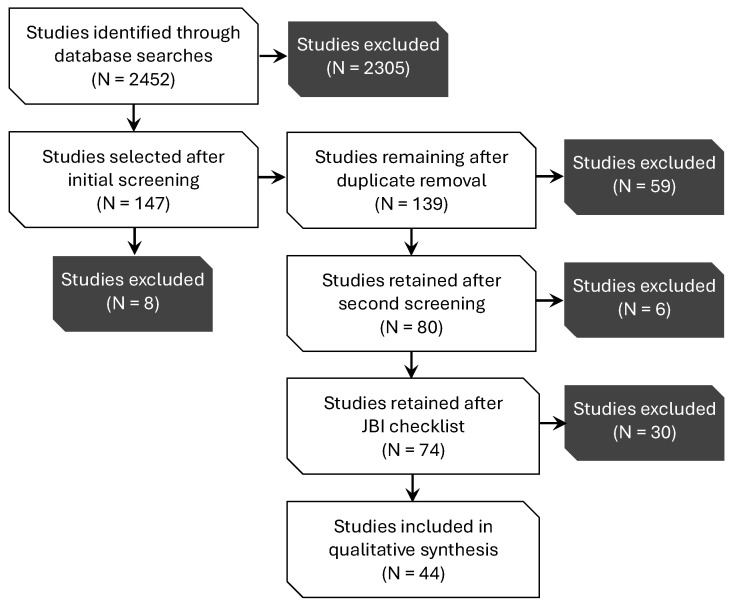
Workflow diagram of the scoping literature review.

The full protocol for this scoping review, including eligibility criteria, search strategy, and data extraction methods, has been prospectively registered and is publicly available in the Open Science Framework (https://osf.io/zhva6; https://doi.org/10.17605/OSF.IO/3NMTW, accessed on 18 September 2025). The completed PRISMA-ScR checklist is provided as Supplementary Material (Table S1). By utilizing a strategic combination of keywords applied to article titles published between 2019 and 2024, the initial search yielded 2452 records, of which 147 were preliminarily selected for further evaluation. Following the removal of duplicate entries, 146 unique studies remained (Figure 1 and Table 2). Eligibility criteria included publication in peer-reviewed journals with an impact factor exceeding 1, as well as relevance verification through a targeted assessment of titles to ascertain alignment with the study’s research questions. To strengthen the transparency and reproducibility of SLR, two reviewers independently screened the titles and abstracts of all retrieved records. Any discrepancies in study selection decisions were resolved through discussion, and when necessary, by consulting a third reviewer to reach consensus.

Subsequently, these studies were classified into nine thematic categories to support a structured screening and relevance assessment. Of the 139 articles assessed for eligibility, 59 were excluded based on predefined relevance criteria, resulting in 80 articles retained for full-text review, as detailed in Table 3. The thematic categories were defined as follows: (1) NbS Benefits, focusing on studies reporting physical, mental, or organizational outcomes; (2) Certifications, referring to frameworks like WELL or LEED in evaluating or supporting NbS integration; (3) Human-Nature (HxN) Connection, addressing theoretical or experiential studies on the psychological or sociological dimensions of nature interaction; (4) Education, encompassing studies applying NbSs in academic settings; (5) Occupational, including direct workplace applications and empirical workplace case studies; (6) Urban Planning, addressing macro-level implementations such as ecological corridors and city-scale strategies; (7) Review, comprising existing systematic, scoping, or narrative reviews on NbSs; (8) Virtual Reality (VR), investigating immersive or simulated nature environments in occupational or built settings; and (9) Policy and Governance, focused on institutional, regulatory, or policy frameworks that facilitate or hinder NbS implementation.

Thereafter, the titles and abstracts of the shortlisted articles were screened to evaluate their relevance against predefined inclusion criteria. The selection parameters prioritized studies that examined the benefits of NbSs across diverse operational contexts and occupational settings (Table 3). Following this preliminary screening, the selected articles were critically appraised using the Joanna Briggs Institute (JBI) Checklist to ensure the methodological rigor, reliability, and validity of the findings.

To further address potential sources of bias, a structured risk of bias assessment was conducted. In addition to the JBI Checklist, a custom evaluation grid was applied, incorporating five key dimensions: methodological clarity, sample representativeness, control of confounding factors, strength of evidence, and alignment with the study’s research questions. Each study was appraised along these dimensions to determine its evidentiary weight in the synthesis. Furthermore, qualitative indicators of bias, such as publication bias, reporting bias, and funding source transparency, were evaluated to contextualize potential distortions. Studies flagged for high risk of bias were retained when offering unique insights but were weighted cautiously in interpretation. This approach reinforces the validity of the synthesis while maintaining inclusivity of emerging or underrepresented perspectives.

During the screening process, studies were retained based on their alignment with the following guiding question: Does the article examine or substantiate the application of NbSs in the integrated management of quality, environmental sustainability, occupational health, and workplace hygiene? This selection process yielded 80 articles for full-text review (Table 3 and Table 4), classified as specified in Table 5.

After applying the JBI critical appraisal criteria, six additional articles were excluded, resulting in 39 studies for the final SLR. A structured critical appraisal process, alongside the JBI checklist, assessed methodological robustness and relevance using a custom evaluation grid covering five dimensions: methodological clarity, sample representativeness, confounding control, evidence strength, and alignment with research questions. A qualitative risk of bias assessment also addressed publication and reporting biases, and funding transparency. Studies with high bias potential were interpreted cautiously to ensure balanced evidence representation.

Borderline articles (Table 6 and Table 7), those that contributed unique insights despite limitations in methodological rigor, were included in the synthesis with caution. While they were not excluded, their findings were weighted less heavily in drawing core conclusions. These articles were used to contextualize emerging trends, highlight underexplored areas, or suggest directions for future research, rather than to substantiate the primary outcomes. Insights from borderline articles were transparently referenced and were not allowed to disproportionately influence overarching interpretations regarding the efficacy of NbSs in occupational environments.

## 4. Results and Discussion

### 4.1. Research Landscape and Emerging Trends

The comprehensive analysis of the selected studies revealed the multifaceted impacts of NbSs on human health and psychological well-being, as well as their influence on organizational productivity and strategic planning for environmental management.

**Figure 2:**
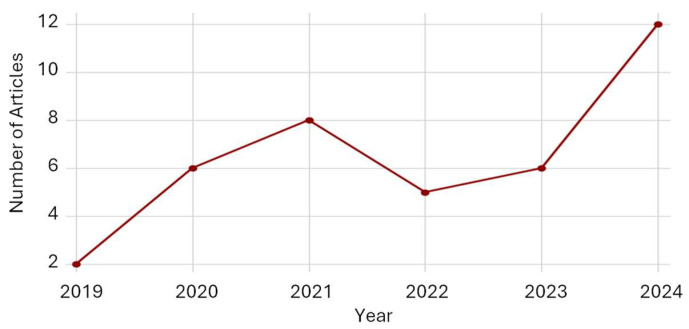
Temporal distribution of reviewed articles.

Within these overarching domains, a series of additional subthemes emerged, which are systematically delineated and synthesized in the subsequent tables for clarity and comparative analysis. Regarding the temporal distribution of the 39 reviewed articles, a consistent increase is evident from 2019 through 2021, reflecting the growing global interest in NbSs and biophilic design within occupational settings (Figure 2).

After this initial growth, the number of publications declined in 2022, likely reflecting the residual effects of pandemic-related disruptions and shifts in research funding priorities. A modest recovery followed in 2023, culminating in a pronounced surge in 2024. This sharp increase suggests a renewed momentum in empirical research and publication, possibly linked to the consolidation of post-pandemic recovery agendas, technological advancements such as immersive virtual nature interventions, and the expanding integration of environmental psychology into architectural and urban design practice. The strong upward trajectory in 2024 highlights the intensifying academic and practical relevance of NbSs as foundational strategies for promoting health, well-being, and sustainability in workplace environments.

**Figure 3:**
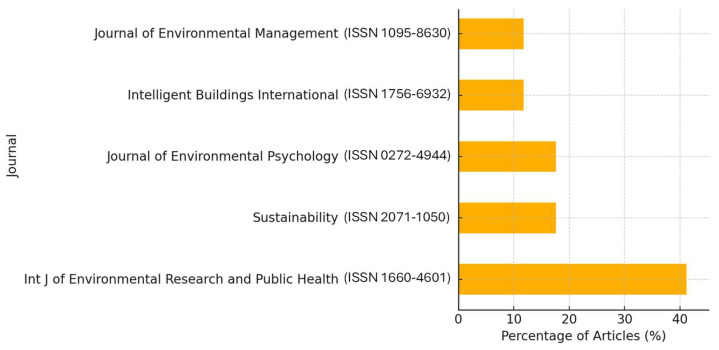
Top journals publishing research on NbSs and workplace well-being.

Figure 3 presents the five most frequent journals contributing to the literature on NbSs and workplace well-being. The Journal of International Journal of Environmental Research and Public Health stands out as the leading publication venue, underscoring the field’s intersection with public health and occupational wellness. Closely following is Sustainability, which highlights the growing integration of sustainable design principles within workplace environments and well-being frameworks.

The Environmental Psychology also features prominently, reflecting its strong focus on the psychological and behavioral dimensions of human–environment interactions in built settings. Intelligent Buildings International and Journal of Environmental Management round out the top five, each contributing a meaningful share of publications that emphasize environmental science, intelligent design, and the optimization of workplace conditions through NbSs.

Together, these journals demonstrate a clear interdisciplinary orientation, bridging psychology, sustainability science, environmental health, and architectural innovation to support the evolving NbSs research agenda.

**Figure 4:**
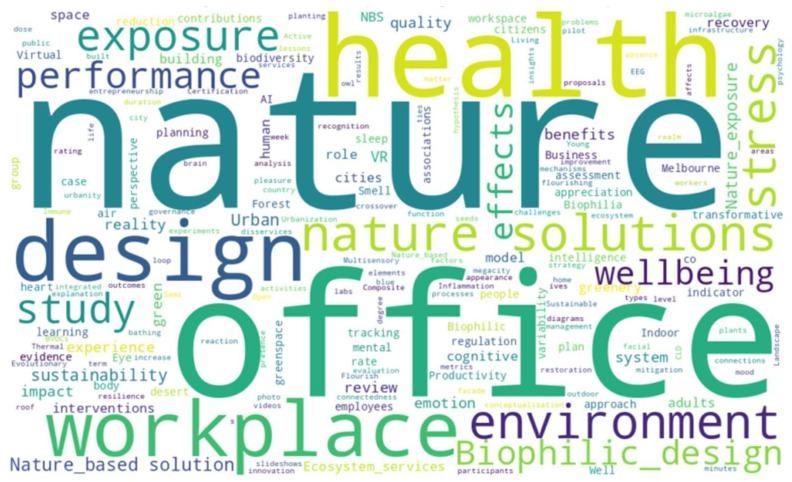
Keyword co-occurrence word cloud of reviewed articles.

The word cloud illustrates the most frequent terms derived from both keywords and nouns extracted from article titles, offering a visual overview of the dominant themes in the reviewed literature (Figure 4). The prominence of terms such as “nature,” “health,” “office,” “design,” and “workplace” underscores the core research focus on the relationship between natural environments and human well-being within professional and built settings.

Phrases like “Nature based,” “Biophilic design,” “Nature Exposure,” and “Solutions” reflect the increasing adoption of NbSs in workplace design. The strong presence of “stress,” “environment,” “performance,” and “recovery” points to ongoing investigations into how exposure to nature can mitigate stress and enhance cognitive and emotional outcomes. Meanwhile, “VR” and “virtual” indicate a technological frontier, where immersive tools are being explored as proxies for real-world nature experiences. Overall, the diversity of terms, spanning health, psychology, design, ecology, and digital technologies, demonstrates the field’s interdisciplinary character and reinforces the importance of integrated approaches to advancing research and practice in NbSs for workplace health promotion.

**Figure 5:**
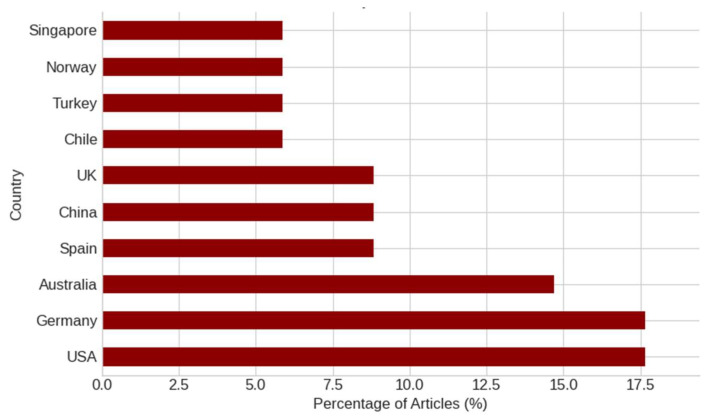
Contributing countries to the NbSs and workplace well-being literature.

Figure 5 highlights the leading countries contributing to the research landscape on NbSs and workplace well-being. Germany, the USA, and Australia emerge as the most prolific contributors, reflecting strong national investments in sustainable building design, workplace wellness, and interdisciplinary research across environmental psychology, public health, and architecture. Spain, China, and Uk also demonstrate significant outputs, indicating growing international engagement in NbS applications within workplace contexts.

The geographical diversity represented, spanning Europe, North America, and the Asia-Pacific, suggests that the NbS discourse is garnering global interest. Nonetheless, the concentration of contributions from high-income countries remains apparent. This highlights the importance of expanding research efforts in underrepresented regions, particularly in Africa and Latin America, to foster more inclusive and globally relevant insights into how NbSs can promote health and well-being in work environments

**Figure 6:**
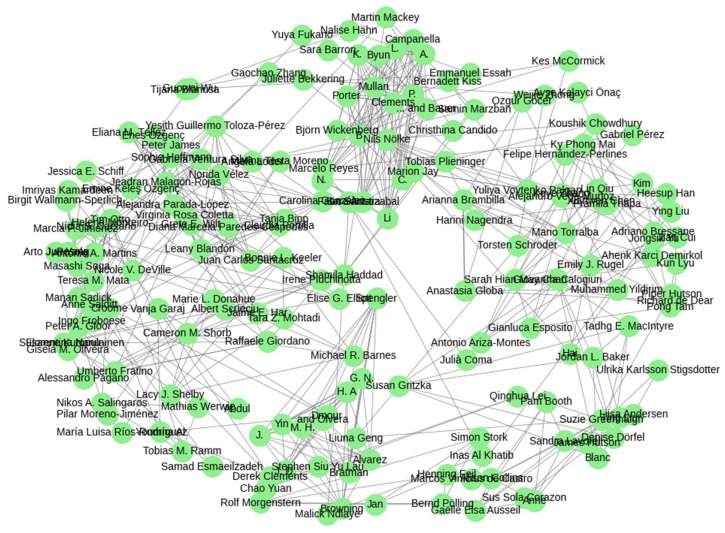
Collaboration network in NbSs and workplace well-being research.

The author collaboration network reveals the structure of co-authorship relationships within the literature on NbSs and workplace well-being (Figure 6). The network is relatively fragmented, with numerous small clusters and a low density of cross-institutional or transnational collaborations. While several tight-knit groups of researchers are visible, often centered around institutional or project-based collaborations, few large, integrated hubs emerge.

This pattern reflects the interdisciplinary yet still emerging nature of the field, where studies are frequently produced within disciplinary silos (e.g., environmental psychology, architecture, public health) or national contexts. Increasing cross-disciplinary and international collaboration could accelerate knowledge integration, methodological innovation, and the development of more robust and generalizable evidence on the effectiveness of NbSs for occupational health. These findings point to the importance of fostering collaborative networks and research consortia to advance this promising but still nascent field of inquiry.

**Figure 7:**
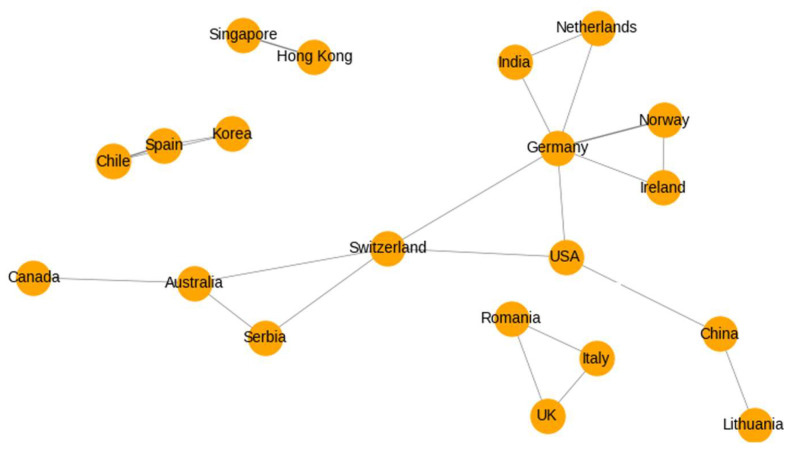
International collaboration network among countries.

The country collaboration network highlights the international connectivity within the literature (Figure 7). While several inter-country partnerships are evident, particularly among European nations and between Europe, North America, and Australia, the overall structure remains fragmented. Notable bilateral collaborations include links between Germany and multiple partners (e.g., USA, Norway, Netherlands), as well as between UK, Italy, and Romania. However, many countries appear as isolated nodes or connected through only a single partnership, suggesting that transnational research networks in this domain are still relatively underdeveloped. The absence of strong hubs or dense clusters reflects the emergent and interdisciplinary nature of this field.

### 4.2. NbS Benefits on Workplace

The findings indicate notable implications for occupational environments, underscoring the contribution of NbSs to fostering healthier workplaces and enhancing overall productivity (Table 8).

#### 4.2.1. Psychophysiological Benefits

**Figure 8:**
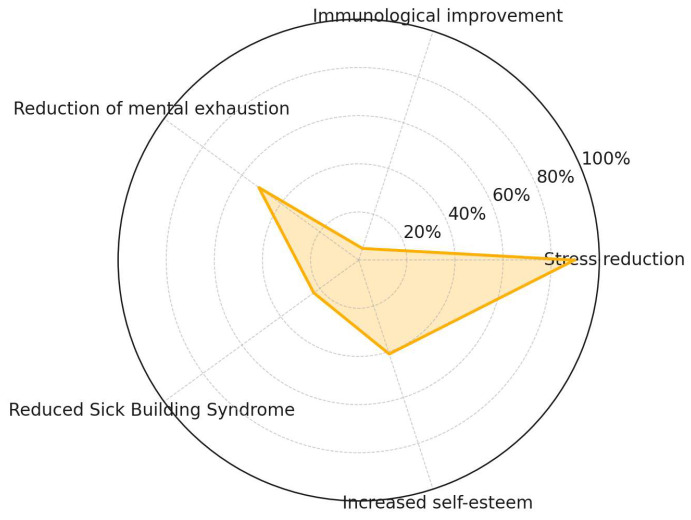
NbS psychophysiological benefits.

A substantial body of evidence demonstrates that NbSs confer psychophysiological benefits within occupational environments (Figure 8). Stress reduction emerges as the most consistently reported outcome, cited in 89.7% of the reviewed studies. In contrast, outcomes such as immunological improvement (5.1%) and reduced sick building syndrome (23.1%) appear less frequently in the literature. Reduction in mental exhaustion is documented in 51.3% of studies, while increased self-esteem is noted in 41.0%. These findings indicate a strong empirical emphasis on subjective psychological responses, with comparatively limited investigation into physiological or clinically measurable health indicators.

One of the most consistently reported outcomes in the literature is stress reduction [29,35]. In the “Second Home” case study, Al-Dmour et al. [29] demonstrated that biophilic design significantly improved occupants’ well-being and productivity. However, they emphasized that vegetation used solely for aesthetic purposes was insufficient. Stress mitigation and overall satisfaction were more effectively achieved when biophilic elements were integrated with environmental quality factors such as air quality, thermal comfort, and acoustics. This suggests that NbSs contribute more reliably to stress reduction in the workplace when embedded within a holistic design strategy rather than applied superficially. Similarly, Barron and Rugel [35] noted that a lack of meaningful nature contact among young adults can weaken psychological resilience and diminish engagement in restorative behaviors. Their framework, which emphasizes order, diversity, and seclusion in urban greenspaces, suggests that stress relief depends not only on the presence of natural features but also on the quality and accessibility of NbSs.

Closely related is the reduction in mental exhaustion, particularly in cognitively demanding environments. Several studies have documented that restorative exposure to natural stimuli, whether physical (such as indoor plants) or virtual (such as immersive digital environments), reduces negative affect and stress while supporting recovery from mental fatigue [37,47]. Chan et al. [37] found that virtual forest walks improved mood and reduced stress as indicated by heart rate measurements, suggesting that digital NbSs can effectively substitute real environments when access to nature is limited. Lyu et al. [47] expanded this perspective by showing that thermal comfort and adaptive options in semi-outdoor workplaces significantly contributed to psychological restoration. Their experiment revealed that the ability to choose between sunlight and shade enhanced attention restoration, stress recovery, and mood. These findings underscore the importance of non-visual sensory pathways, particularly thermal perception, in reducing mental fatigue. Overall, NbSs alleviate exhaustion not only through visual engagement with greenery but also by activating multisensory restorative mechanisms.

Enhanced cognitive performance, especially in terms of concentration and attentional capacity, has also been widely reported [27,32]. Aly [27] introduced the Biophilic Design Criteria for Productivity (BDCP), arguing that reconnecting employees with natural elements can mitigate stressors such as high workloads, competition, and performance pressure, thereby fostering environments more conducive to focus and efficiency. Although primarily conceptual, this framework links biophilic principles to architectural strategies, emphasizing the role of evidence-based design in translating nature’s restorative potential into measurable performance outcomes. Complementing this, Aristizabal et al. [32] presented experimental evidence showing that multisensory biophilic interventions enhanced executive function, reduced stress, and increased workplace satisfaction. Together, these findings suggest that biophilic design strengthens neurocognitive functioning through both theoretical frameworks that inform architectural practice and empirical validation in real-world and laboratory settings.

A notable yet less frequently highlighted benefit is immunological improvement, as reported by Paredes-Céspedes et al. [49], pointing to potential systemic health advantages from sustained nature exposure. Their systematic review found that, although the evidence base remains limited, some randomized clinical trials reported reductions in cortisol levels and lower stress responses among participants engaged in nature-based therapeutic interventions. These outcomes indicate possible pathways for immune system enhancement. However, results across studies were heterogeneous, with several showing no significant effects on anxiety and depression measures, highlighting the need for more rigorous, biomarker-driven research.

Additionally, several studies reported reductions in Sick Building Syndrome (SBS) symptoms such as headaches, respiratory irritation, and general discomfort following the implementation of NbSs [36,39]. Evidence from certified open-plan offices in Australia showed that environments incorporating active and biophilic design principles, including those rated under the WELL standard, scored higher on perceived health, productivity, thermal comfort, and spatial quality compared to conventional offices [36]. These improvements were strongly linked to the mitigation of environmental stressors such as poor air circulation, noise, and inadequate ergonomics, all commonly associated with SBS. Similarly, Demirkol and Önaç [39] emphasized that integrating biophilic elements such as natural lighting, evolving organic materials, and views of nature into office spaces supports healthier and more restorative environments. Their findings suggest that these design strategies not only enhance psychological well-being but also reduce physical discomforts characteristic of SBS.

An often overlooked yet recurring outcome is the improvement of self-esteem associated with natural elements in the workplace. Ausseil et al. [33], using a participatory framework in New Zealand, found that non-material and regulatory contributions from nature, such as environmental quality, social connections, and health, are directly linked to multiple dimensions of human well-being. Their findings highlight the importance of recognizing and incorporating nature–well-being relationships into policy and workplace design to strengthen psychological stability and foster a sense of individual value and belonging. Complementarily, Wickenberg et al. [55] examined two large-scale NbS projects in Sweden and demonstrated that transformative learning, enabled through cross-sector collaboration and citizen engagement, is essential to realizing the full health and well-being potential of NbSs. This perspective underscores that gains in self-esteem are not solely the result of individual exposure to nature but also emerge from collective processes that promote empowerment, participation, and social cohesion.

#### 4.2.2. Psychosocial and Motivational Outcomes

**Figure 9:**
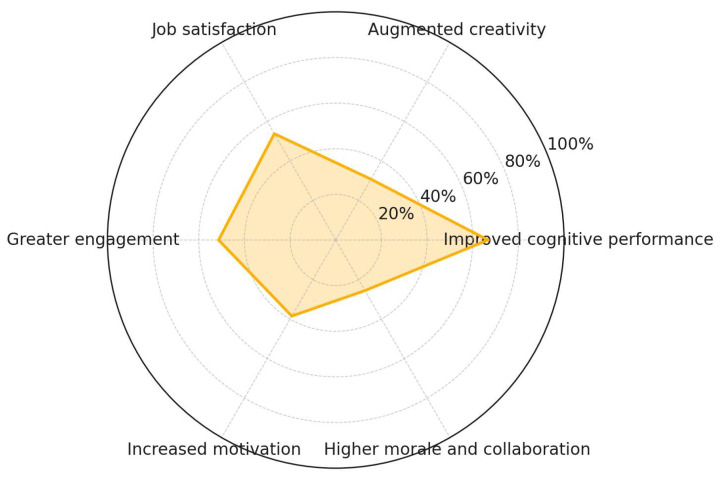
NbS psychosocial and motivational outcomes.

Among psychosocial and motivational outcomes, improved cognitive performance is the most prevalent, reported in 66.7% of the reviewed studies. Other frequently cited outcomes include job satisfaction (53.9%) and greater engagement (51.3%), followed by increased motivation (38.5%) and augmented creativity (30.8%). Higher morale and collaboration were documented in 25.6% of studies. This reflects a relatively balanced acknowledgment of the positive influence of nature-based interventions on occupational well-being. However, it also underscores the need for broader and more systematic empirical coverage of these constructs to substantiate their role in workplace health and performance (Figure 9).

Job satisfaction is among the most consistently reported outcomes, with strong empirical support across diverse settings. Studies suggest that contact with nature fosters affective commitment and perceived meaning in work, contributing to greater emotional well-being and organizational attachment. This is further reinforced by the consistent association between natural features and greater engagement, a construct encompassing psychological investment, enthusiasm, and task involvement [8,48].

Exposure to natural environments has been shown to enhance motivation, foster positive affect, and support sustained task engagement [10]. In parallel, studies highlight that nature-based solutions also stimulate broader forms of engagement, collaboration, and collective participation within organizational and governance contexts [55]. These findings align with broader theoretical frameworks on the psychological effects of greenery [40], which highlight the motivational power of nature-rich environments in sustaining performance and reducing psychological withdrawal.

NbSs also appear to positively influence morale and collaboration, promoting social connection and team cohesion. Several studies report that biophilic environments foster informal interactions, trust-building, and collective problem-solving [35,52]. In turn, this contributes to healthier team dynamics and a reduction in social isolation, an increasingly relevant concern in hybrid and post-pandemic work models.

Although somewhat less studied, enhanced cognitive performance has emerged as a salient benefit of nature-integrated environments. Enriched sensory stimuli, diverse textures, and restorative spatial layouts are associated with improved attention and reduced mental fatigue [33,41,56]. These effects are particularly valuable in design-intensive and cognitively flexible work contexts.

#### 4.2.3. Organizational and Economic Findings

**Figure 10:**
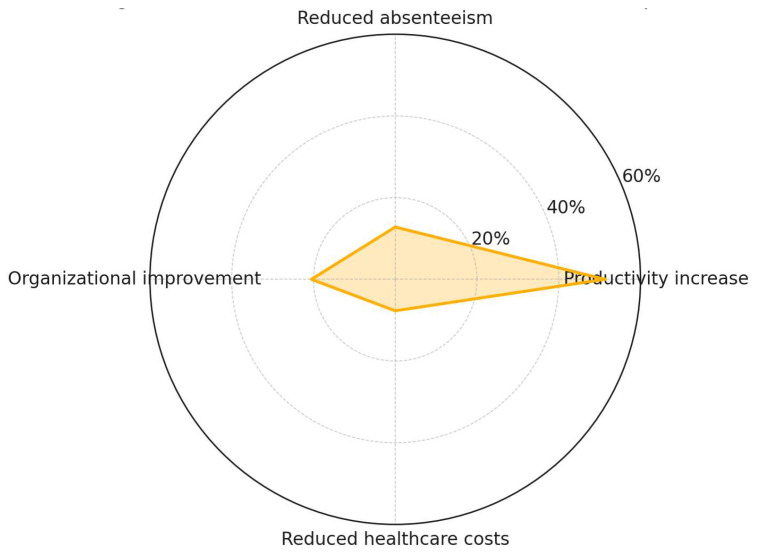
NbS organizational and economic findings.

Figure 10 indicates that productivity increase is the most frequently reported outcome, appearing in 51.3% of the reviewed studies. Organizational improvement (20.5%) is addressed to a moderate extent, while reduced absenteeism (12.8%) and reduced healthcare costs (7.7%) are the least frequently reported. The limited representation of direct economic indicators highlights a persistent gap in the quantification of financial returns associated with NbSs in workplace contexts. This underscores the need for future research to incorporate cost-effectiveness analyses, standardized performance metrics, and longitudinal tracking to evaluate organizational outcomes with greater precision.

Productivity increase emerges as the most consistently documented organizational outcome, reported across a wide range of workplace typologies and intervention scales. Within participatory approaches to NbSs, Coletta et al. [38] emphasize that co-design processes generate multiple co-benefits, including those directly linked to organizational efficiency and performance. By integrating stakeholders’ perspectives into the planning and evaluation phases, tools such as causal loop diagrams and performance assessment frameworks help identify solutions that advance both social and environmental goals while simultaneously strengthening systemic productivity. Complementarily, Ramm et al. [50] provide experimental evidence that biophilic design features in built environments evoke more positive emotions and correlate positively with productivity and team engagement. Their findings, based on facial emotion recognition and sentiment analysis, demonstrate that natural elements embedded in architectural spaces enhance affective responses while reinforcing collective motivation, thereby consolidating the link between biophilia, well-being, and organizational performance.

In parallel, several studies report reductions in absenteeism, a critical indicator tied to both physical health and psychological resilience. Sadick and Kamardeen [10], for example, show that workplace exposure to natural elements improves health, stress reduction, and coping capacity, with direct implications for organizational sustainability. Their systematic review highlights that incorporating both indoor and outdoor nature exposure into workplace design maximizes employee benefits, contributing not only to enhanced well-being but also to operational continuity through lower rates of sickness-related absences. These findings suggest that nature-integrated interventions foster worker satisfaction while strengthening workforce resilience.

With respect to financial outcomes, reduced healthcare costs have been documented in studies monitoring wellness metrics and linking declines in absenteeism to lower medical claims and expenditures. Stork et al. [52] demonstrate that integrating NbSs into organizational models generates value not only through environmental and social contributions but also through economic returns. Their NbS Business Model Canvas (NbS BMC) provides a framework for incorporating health-related gains, including the reduction in workplace stressors and healthcare burdens, into business strategies. This perspective underscores that the financial benefits of NbSs are inseparable from their broader environmental and social co-benefits, reinforcing their role in lowering organizational costs while improving employee well-being.

Organizational improvement as an overarching outcome encompasses strategic gains such as stronger governance, enhanced innovation capacity, and alignment with sustainability objectives. Yu et al. [58] showed that nature-friendly environments in the hospitality sector significantly reduced employee burnout while enhancing job satisfaction and performance. These individual-level improvements translated into broader organizational benefits, including more effective human resource management and stronger alignment with strategic goals. By illustrating the link between NbSs and improved workplace performance, this study reinforces the potential of biophilic and eco-friendly environments to strengthen organizational resilience and competitiveness while advancing sustainability-driven management practices.

### 4.3. NbS Challenges and Opportunities

The findings reveal important opportunities and challenges of NbSs to planning and management processes, emphasizing their barriers and potential to support more integrated, adaptive, and sustainable approaches to decision-making (Table 9).

#### 4.3.1. Challenges to Implementing NbSs

**Figure 11:**
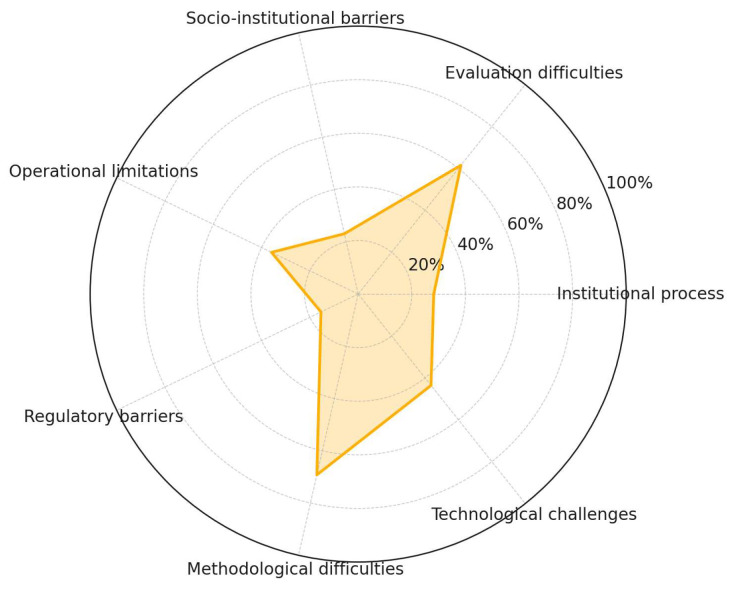
Challenges to implementing NbSs.

Despite their well-documented benefits, Figure 11 illustrates a diverse range of systemic and operational challenges that hinder the implementation of NbSs in occupational settings. Methodological difficulties (69.2%) and evaluation difficulties (61.5%) emerge as the most frequently reported constraints, highlighting persistent gaps in standardized metrics, longitudinal assessment, and interdisciplinary integration. Sociocultural barriers (41.0%), structural limitations (38.5%), and operational limitations (35.9%) are also commonly mentioned, underscoring the contextual and practical complexities of implementation. Financial challenges (33.3%) and institutional process-related barriers (28.2%) reflect the economic and governance hurdles that often restrict adoption, while technological challenges (43.6%) remain a consistent obstacle. Finally, socio-institutional barriers (23.1%) and regulatory barriers (15.4%) represent less frequent but still significant impediments, pointing to the multi-layered nature of these constraints.

Among the most frequently reported constraints are evaluation difficulties, which appear across nearly all reviewed studies. Wallmann-Sperlich et al. [54] highlight this issue in their pilot study on active biophilic office design, where small sample sizes and the short follow-up period limited the ability to generalize results and assess long-term impacts. While the relocation to an “active” biophilic workspace increased standing time among employees, the absence of changes in other work-associated factors illustrates the difficulty of capturing the full scope of biophilic interventions within restricted methodological designs. These limitations underscore the broader challenge of establishing standardized indicators and longitudinal evaluation frameworks, which are essential for demonstrating the sustained organizational and health benefits of NbSs.

Closely related are methodological difficulties, reported in studies such as those by Lyu et al. [47]. Their experimental design, which tested restorative benefits of semi-outdoor workplace environments under distinct thermal scenarios, revealed how variations in thermal perception and adaptive opportunities influenced psychological outcomes. Although the study demonstrated significant associations between thermal pleasure and restorative benefits such as attention restoration and stress recovery, it also exposed challenges in isolating causal effects and accounting for multisensory environmental variables. These methodological constraints illustrate the broader difficulty of designing NbS research that adequately integrates complex environmental factors, while maintaining internal validity and producing findings that are generalizable across diverse workplace contexts.

Sociocultural barriers, such as lack of awareness, cultural resistance, or undervaluation of nature-based approaches, remain a substantial concern, particularly in contexts with limited environmental education or entrenched architectural traditions. Candido et al. [36] show that even in certified office environments, the adoption of active and biophilic design does not always guarantee user satisfaction or behavioral change, as organizational culture and user perceptions significantly mediate outcomes. Similarly, Barron and Rugel [35] demonstrate how young adults often face reduced opportunities to connect with nature in rapidly urbanizing environments, a condition that risks perpetuating weaker environmental bonds and lower engagement in pro-environmental behaviors.

Socio-institutional barriers and institutional process challenges also impede implementation. Ausseil et al. [33] emphasize that while nature’s contributions to well-being are increasingly acknowledged, institutional inertia and fragmented governance structures limit the integration of NbSs into long-term policy frameworks. Similarly, Wickenberg et al. [55] demonstrate that effective NbS implementation depends on transformative learning processes, yet these are often constrained by limited stakeholder participation, rigid institutional arrangements, and insufficient reflexive governance. Together, these studies highlight that without cross-boundary collaboration, action-oriented knowledge production, and continuity in governance, NbS projects risk remaining fragmented pilot initiatives rather than being scaled into durable systemic change.

At the practical level, several studies cite structural limitations as significant barriers to implementation. Thapa et al. [53] found that while tree cover and green infrastructure were positively associated with well-being, their distribution varied greatly along the rural–urban gradient of Bengaluru. This uneven access highlights a structural constraint: in dense urban centers, limited spatial availability and competing land-use priorities restrict the integration of NbSs, whereas in peri-urban or rural areas, natural elements are more abundant but often undervalued in planning. Moreover, their findings underscore that well-being outcomes are mediated not only by the presence of greenery but also by social factors such as community activities and relationships. This suggests that structural barriers to NbS implementation extend beyond the physical fabric of buildings and cities, encompassing the interplay between spatial constraints, urbanization pressures, and the social infrastructure that supports equitable access to nature.

Technological challenges, particularly related to simulation, integration, and accessibility, are also documented. Chan et al. [37] demonstrated that while VR simulations of natural environments significantly reduced stress and improved affective states among both young adults and senior citizens, limitations persist in terms of accessibility and adaptability. The study underscored that VR-based NbSs require adequate technological infrastructure and user familiarity, which may not be uniformly available across organizations. Moreover, challenges arise in integrating these digital interventions into daily occupational routines, as extended VR exposure can lead to discomfort and reduced practicality in workplace settings. Thus, while virtual environments offer a promising avenue for expanding access to nature in constrained contexts, technological and ergonomic barriers remain central obstacles to their broader adoption.

Regulatory barriers, though mentioned less frequently, emerge as impediments where planning codes, environmental licensing, or construction standards do not yet accommodate NbSs. Ausseil et al. [33] highlight that although ecosystem services are increasingly recognized within sustainability frameworks, there is still a misalignment between scientific evidence and regulatory mechanisms. Their findings demonstrate that existing classification systems and policy instruments often fail to capture the non-material contributions of nature to human well-being, resulting in gaps when translating NbS benefits into enforceable regulations. This regulatory lag restricts the institutionalization of NbSs, delaying their systematic adoption across occupational and urban environments.

Financial challenges, including initial capital costs, limited funding mechanisms, and unclear cost–benefit calculations, remain significant in both public and private sector implementations. Aly [27] addressed this issue by proposing the BDCP, which emphasize the necessity of integrating cost-effectiveness into workplace planning. The study underscores that while biophilic interventions are often perceived as aesthetic enhancements, they must be systematically framed as productivity-driven investments to secure financial legitimacy. Without clear metrics linking biophilic design to measurable returns such as reduced stress, enhanced motivation, and increased output, organizations are less inclined to allocate resources. Thus, economic framing and evidence-based design principles are crucial to overcoming the financial hesitation surrounding NbSs adoption.

#### 4.3.2. Opportunities to Implement NbSs

**Figure 12:**
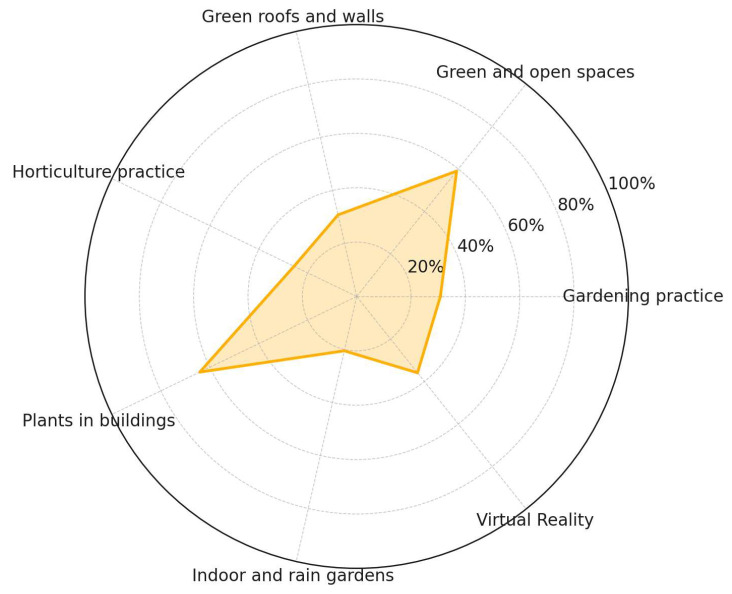
Opportunities to implement NbSs.

While the implementation of NbSs face notable challenges, the reviewed literature also reveals a diverse set of opportunities that facilitate their integration into occupational environments (Figure 12). Several high-frequency interventions have already been successfully applied or proposed in workplace contexts. Indoor plants (64.1%) emerged as the most frequently cited opportunity, reflecting both their feasibility and their restorative potential as biophilic interior elements. Green and open spaces (59.0%) closely followed, representing nature-integrated architectural strategies with strong environmental and psychological co-benefits. Virtual reality (33.3%) also stands out as a promising innovation for delivering nature exposure in spatially constrained workplaces. In addition, gardening practices (30.8%) and green roofs and walls (30.8%) offer accessible strategies for ecological integration, while horticultural interventions (25.6%) and indoor or rain gardens (20.5%) provide complementary approaches that promote active engagement with nature. This diversity of interventions highlights the adaptability of NbSs and underscores their potential scalability across a wide range of workplace typologies and resource contexts.

Gardening and horticultural practices are particularly notable as highly accessible and participatory forms of NbSs, fostering direct human–nature interaction and psychological restoration. Barron and Rugel [35] show that structured engagement with urban greenspaces strengthens social ties, encourages pro-environmental behaviors, and supports mental health among young adults. Translated into occupational contexts, gardening and horticulture initiatives function not only as aesthetic enhancements but also as mechanisms for building social cohesion and promoting mental well-being in workplaces. Complementarily, Chan et al. [37] demonstrate that virtual simulations of natural environments can reproduce restorative benefits, including stress reduction and enhanced connectedness to nature, even in settings where physical gardening is not feasible. Taken together, these findings indicate that both hands-on horticultural practices and their digital extensions offer scalable opportunities for NbS integration in diverse occupational environments.

Green and open spaces remain the most consistently cited spatial opportunities, offering multifunctional benefits such as environmental regulation, social connection, and recreational value. Ausseil et al. [33] emphasize that recognizing the role of nature in human well-being requires robust evidence and systematic monitoring to inform long-term sustainability strategies. Their participatory framework illustrates how non-material contributions of ecosystems, such as fostering social ties and improving environmental quality, are central to well-being. Applied to occupational settings, these insights suggest that integrating green and open spaces into workplaces can advance ecological goals while simultaneously enhancing social connectedness and improving the perceived quality of the work environment.

Green roofs and rain gardens, although requiring more complex structural planning, deliver essential ecosystem services including stormwater management, thermal regulation, and habitat support. Aristizabal et al. [32] demonstrate that multisensory biophilic environments, combining visual, auditory, and other sensory stimuli, significantly enhance cognitive performance, reduce stress, and increase environmental satisfaction. Extending these findings, green roofs and rain gardens can be understood not only as ecological infrastructures but also as restorative spaces that engage multiple senses. By contributing to both environmental performance and human well-being, these interventions align closely with sustainable workplace strategies aimed at optimizing comfort, productivity, and resilience.

A widely recognized yet underutilized opportunity lies in the incorporation of plants within buildings, ranging from potted flora to vertical green walls and biophilic architectural integration. Yin et al. [15] provide experimental evidence that offices enriched with biophilic features, even when simulated in virtual reality, consistently lowered physiological stress indicators and improved creativity. Extending this research, Yin et al. [57] compared virtual exposure to various environmental conditions and found that even arid landscapes facilitated stress recovery, although green environments yielded stronger restorative effects. Together, these findings confirm that indoor vegetation functions not merely as decoration but as an active contributor to stress reduction, creativity, and overall well-being. Strategically embedding vegetation into office design allows workplaces to harness these restorative benefits while simultaneously supporting cognitive performance.

Emerging technological approaches, particularly virtual reality, represent a promising frontier for NbSs. Yildirim et al. [56] discuss that multisensory biophilic experiences in virtual environments significantly enhance cognitive performance, reduce stress, and improve mood states in workplace settings. Their results reveal that while visual components remain fundamental, olfactory stimuli play a critical and previously overlooked role in shaping restorative outcomes. This suggests that VR-enabled NbSs, when designed as multisensory experiences, can more effectively replicate the benefits of direct nature contact compared to visual simulation alone. Such interventions provide scalable opportunities for organizations to deliver restorative experiences in dense urban areas or spatially constrained workplaces, positioning VR as a versatile tool for advancing occupational well-being.

## 5. Limitations and Future Research

This scoping review offers contributions to the emerging field of NbSs in occupational settings. Nonetheless, some limitations must be acknowledged. First, despite the search strategy applied, the review was limited to two major databases, Scopus and Web of Science. This may have inadvertently excluded relevant studies indexed in other repositories, specialized databases, or grey literature sources. Second, the focus on English-language, peer-reviewed journals with an impact factor greater than one introduces potential publication and language bias. While this ensures scientific rigor, it may marginalize valuable research outputs from non-English-speaking scholars or from regions where NbSs research is emergent but less likely to be published in high-impact outlets. Third, although a critical appraisal was conducted using both the JBI Checklist and a customized evaluation matrix, the heterogeneity across included studies, in terms of design, methodological quality, and outcome measures, constrains the ability to synthesize consistent effect sizes or draw causal inferences. This methodological diversity, while contributing to the breadth of the synthesis, limits the depth of comparative analysis. Fourth, the evidence base is predominantly drawn from high-income countries, with limited representation from African, Latin American, and Southeast Asian contexts. This geographical imbalance may affect the generalizability of the findings, as socioeconomic, climatic, and regulatory differences can influence both the feasibility and effectiveness of NbS interventions.

To address these limitations, future research should broaden its scope to include underrepresented regions and workplace typologies, including informal economies, hybrid work settings, and industries with elevated environmental risks. Longitudinal studies employing physiological markers and standardized performance metrics are essential to strengthen causal attributions. Greater methodological harmonization, especially in differentiating active versus passive nature exposure, would enhance cross-study comparability.

In addition, emerging technologies such as virtual reality and AI-driven adaptive design warrant further investigation for their potential to deliver nature-based experiences in spatially or logistically constrained environments. Empirical analyses of cost-effectiveness and return on investment across different organizational sizes and sectors are also urgently needed to support decision-making and mainstream adoption. Lastly, participatory, transdisciplinary research approaches should be prioritized. Involving end-users, designers, and facility managers in co-creating and evaluating NbSs can enhance stakeholder engagement and implementation success. Integration with certification frameworks like WELL and LEED may further institutionalize NbSs within corporate QEHS strategies and policy frameworks.

## 6. Conclusions

This scoping review mapped the benefits, challenges, and implementation pathways of NbSs in workplace environments, underscoring their transformative potential in advancing employee health, psychological well-being, environmental quality, and organizational sustainability. Across the reviewed literature, NbSs consistently revealed promising outcomes, including stress reduction, enhanced cognitive function, increased job satisfaction, and decreased absenteeism, factors that collectively contribute to the development of healthier, more productive work environments. However, despite these benefits, the integration of NbSs into QEHS management systems remains limited by several persistent barriers. These include methodological inconsistencies, high initial investment costs, technological limitations, and institutional resistance to change. Addressing these challenges requires the adoption of interdisciplinary frameworks, the development of standardized evaluation metrics, and the implementation of supportive policy mechanisms that facilitate structured and scalable adoption.

Future research should emphasize longitudinal designs and incorporate physiological biomarkers to substantiate causal relationships between NbS interventions and workplace outcomes. The adoption of standardized outcome measures will also improve comparability across studies. Moreover, the use of immersive technologies, such as virtual reality, offers innovative opportunities to deliver restorative experiences in spatially constrained or urbanized work environments. Equally important is the integration of participatory co-design processes that engage key stakeholders, such as employees, facility managers, and design professionals, in the development and evaluation of NbSs. Aligning implementation efforts with established certification systems, including WELL and LEED, can enhance legitimacy, facilitate institutional uptake, and promote scalability.

## Figures and Tables

**Table 1:** Database Search Strategy.

Category	Term/Combination	Operator
Nature	“Nature-based solutions”, “Biophilic Design”, “Nature Exposure”	OR
Results	“Environmental Quality”, “Health”, “Safety”, “Productivity”	OR
Tools	“Nature-based solutions”	OR
Filters	2019–2024, Impact Factor > 1, Search in: Article Title, Abstract, Keywords	AND

**Table 2:** Search strategy.

Term/Combination	Search	Retained
“Nature-based solutions” OR “Biophilic Design” OR “Nature Exposure” AND “Environmental Quality”	1221	44
“Nature-based solutions” OR “Biophilic Design” OR “Nature Exposure” AND “Health”	812	69
“Nature-based solutions” OR “Biophilic Design” OR “Nature Exposure” AND “Safety”	242	6
“Nature-based solutions” OR “Biophilic Design” OR “Nature Exposure” AND “Productivity”	177	25
Total	2452	144
Total after duplicate removal		139

**Table 3:** Retained and reasons of exclusion.

Category	Qty.	Retained	Reasons of Exclusion
NbS Benefits	43	30	Focused on non-occupational contexts (e.g., public parks, residential areas). Lacked quantitative health/productivity metrics. Addressed mental health outcomes without workplace-specific interventions. Superficially mentioned NbSs without empirical validation.
Certifications	1	1	N/A
HxN Connection	1	1	N/A
Education	3	2	Examined academic performance without workplace relevance.
Occupational	21	16	N/A
Urban Planning	42	3	Prioritized city-scale infrastructure (e.g., green corridors) over workplace design. Focused on policy without practical implementation in occupational settings. Addressed flood management without health or productivity co-benefits.
Review	20	14	Broadly discussed NbSs without occupational health or QEHS alignment. Synthesized evidence on ecosystem services unrelated to workplaces. Lacked critical appraisal of NbS implementation barriers in corporate settings.
VR	8	13	Lacked protocols to translate VR nature exposure into design guidelines.
TOTAL	139	80	

**Table 4:** Predominant methodologies in 80 included articles.

Methodology	%	Application Example
Systematic Reviews	25	Evidence on NbSs and mental health.
Experimental Studies	18	VR or biophilia interventions.
Observational Studies	22	Longitudinal data (e.g., cortisol levels).
Qualitative Studies	12	Interviews on green space perceptions.
Data Modeling/Analysis	23	Bibliometrics, or machine learning.
Total	100	

**Table 5:** Reasons for excluding 6 articles.

Reason	%	Example
Methodological Clarity	33	Undescribed methods.
Unrepresentative Sample	17	15 participants, no demographic diversity.
Confounding Bias	17	No control for socioeconomic factors.
Non-Significant Results	17	Insignificant differences in ROS scale.
Off-Topic	16	Building certifications unrelated to health.
Total	100	

**Table 6:** Justification for Including “borderline” articles.

Inclusion Criterion	%	Contribution
Policy Relevance	30	Supports green infrastructure policies.
Methodological Innovation	25	New tools for psychological assessment.
Emerging Evidence	20	Solutions for polluted urban environments.
Understudied Contexts	15	Highlights nature-access inequalities.
Interdisciplinary Value	10	Integrates architecture and occupational psychology.
Total	100	

**Table 7:** Rigor vs. relevance summary.

Category	%	Details
JBI-Compliant Articles	88	Followed strict methodological criteria.
Justified “Borderline” Articles	12	Included for unique contributions.
Total Articles Analyzed	100	Complete database.

**Table 8:** Findings of NbS benefits on workplace.

	Increased Self-Esteem ^1^	Immunological Improvement ^1^	Reduced Sick Building Syndrome ^1^	Reduction in Mental Exhaustion ^1^	Stress Reduction ^1^	Augmented Creativity ^2^	Greater Engagement ^2^	Higher Morale and Collaboration ^2^	Improved Cognitive Performance ^2^	Increased Motivation ^2^	Job Satisfaction ^2^	Organizational Improvement ^3^	Productivity Increase ^3^	Reduced Absenteeism ^3^	Reduced Healthcare Costs ^3^
Al Dmour et al. [29]			x	x	x		x	x		x	x		x		
Al Khatib et al. [30] ^b^					x				x		x				
Aly [27] ^a^				x	x	x	x	x	x	x	x	x		x	
Andersen et al. [31] ^b^	x	x	x		x										
Aristizabal et al. [32] ^a^					x		x		x		x		x		
Ausseil et al. [33] ^a^	x				x	x			x		x	x	x		
Barnes et al. [34] ^b^	x			x	x				x		x				
Barron and Rugel [35] ^a^	x				x	x	x		x		x		x		
Bressane and Castro [23] ^a^					x		x		x	x	x	x	x	x	
Candido et al. [36] ^a^			x		x		x		x	x	x	x	x	x	x
Chan et al. [37] ^a^				x	x										
Coletta et al. [38] ^a^					x		x						x		
Demirkol and Önaç [39] ^a^	x		x		x	x	x	x	x	x	x		x	x	
Fukano and Soga [40] ^a^	x				x				x						
Gritzka et al. [8] ^b^	x			x	x		x		x	x	x				
Hähn et al. [41] ^b^						x			x			x	x		
Hutson and Hutson [6] ^b^	x		x		x	x	x	x	x	x	x		x		
Jimenez et al. [42] ^b^	x			x	x										
Kumpulainen et al. [43] ^a^				x	x	x	x	x	x	x					
Lei et al. [44] ^a^				x					x				x		
Liu et al. [45] ^b^					x								x		
Liu et al. [46] ^a^	x				x				x						
Lyu et al. [47] ^a^				x	x				x						
Özgenç and Özgenç [48] ^b^					x										
Paredes-Céspedes et al. [49] ^b^		x		x	x										
Ramm et al. [50] ^a^					x		x		x	x	x		x		
Reyes et al. [51] ^b^			x	x	x						x		x		
Ríos-Rodríguez et al. [7] ^b^				x	x	x	x	x	x	x	x				
Sadick and Kamardeen [10] ^b^			x	x	x	x	x		x	x	x		x	x	x
Stork et al. [52] ^a^	x						x	x		x		x	x		x
Thapa et al. [53] ^a^	x			x	x				x						
Wallm-Sperlich et al. [54] ^a^			x	x	x		x		x	x	x		x		
Wickenberg et al. [55] ^a^	x						x	x			x	x			
Yildirim [56] ^a^	x			x	x		x	x	x	x	x		x		
Yin et al. [15] ^a^					x	x			x						
Yin et al. [57] ^a^				x	x	x									
Yu et al. [58] ^a^				x	x						x	x	x		
Zhang et al. [59] ^a^	x			x	x		x		x						
Zhong et al. [12] ^b^	x		x	x	x	x	x	x	x	x	x		x		

^a^ research articles; ^b^ review articles; ^1^ psychophysiological benefits; ^2^ psychosocial and motivational outcomes; ^3^ organizational findings.

**Table 9:** Findings on NbSs challenges and opportunities.

	Evaluation Difficulties ^1^	Financial Challenges ^1^	Institutional Process Barriers ^1^	Methodological Difficulties ^1^	Operational Limitations ^1^	Regulatory Barriers ^1^	Sociocultural Barriers ^1^	Socio-Institutional Barriers ^1^	Structural Limitations ^1^	Technological Challenges ^1^	Plants in Buildings ^2^	Gardening Practice ^2^	Green and Open Spaces ^2^	Green Roofs and Walls ^2^	Horticulture Practice ^2^	Indoor and Rain Gardens ^2^	Virtual Reality ^2^
Al Dmour et al. [29} ^a^											x						
Al Khatib et al. [30] ^b^	x	x	x	x	x		x	x	x	x	x	x	x	x	x	x	
Aly [27] ^a^	x	x						x	x		x	x	x	x	x	x	
Andersen et al. [31] ^b^	x			x							x		x				
Aristizabal et al. [32] ^a^	x			x	x				x	x	x		x	x		x	
Ausseil et al. [33] ^a^	x			x		x	x	x		x		x	x	x	x	x	
Barnes et al. [34] ^b^	x			x								x	x				
Barron and Rugel [35] ^a^	x		x	x	x	x	x		x			x	x	x	x	x	
Bressane and Castro [23] ^a^		x		x	x				x		x			x		x	
Candido et al. [36] ^a^	x						x			x							
Chan et al. [37] ^a^	x	x	x	x	x		x	x	x	x	x	x	x		x		x
Coletta et al. [38] ^a^	x		x	x				x									
Demirkol and Önaç [39] ^a^			x				x				x		x				
Fukano and Soga 40] ^a^	x			x			x				x		x				x
Gritzka et al. [8] ^b^	x			x							x	x	x	x	x		
Hähn et al. [41] ^b^	x				x				x		x						
Hutson and Hutson [6] ^b^		x								x	x	x		x			x
Jimenez et al. [42] ^b^	x			x			x					x	x		x		x
Kumpulainen et al. [43] ^a^				x						x							x
Lei et al. [44] ^a^				x						x	x						x
Liu et al. [45] ^b^	x	x	x					x									
Liu et al. [46] ^a^				x			x			x			x				x
Lyu et al. [47] ^a^				x						x			x				x
Özgenç and Özgenç [48] ^b^	x			x			x										
Paredes-Céspedes et al. [49] ^b^	x	x	x		x	x	x		x	x	x	x	x	x	x	x	
Ramm et al. [50] ^a^	x			x						x	x		x				
Reyes et al. [51] ^b^	x	x	x	x	x	x			x	x	x			x			
Ríos-Rodríguez et al. [7] ^b^				x	x					x	x		x				x
Sadick and Kamardeen [10] ^b^	x	x		x	x				x		x		x	x			
Stork et al. [52] ^a^	x	x	x	x	x		x	x	x	x		x	x		x		
Thapa et al. [53] ^a^	x	x		x			x		x		x		x				
Wallm-Sperlich et al. [54] ^a^	x				x				x	x	x						
Wickenberg et al. [55] ^a^		x	x		x	x	x	x	x								
Yildirim [56] ^a^										x	x						x
Yin et al. [15] ^a^											x						x
Yin et al. [57] ^a^				x			x				x						x
Yu et al. [58] ^a^				x							x		x				
Zhang et al. [59] ^a^				x									x				x
Zhong et al. [12] ^b^	x	x	x	x	x	x	x	x	x		x	x	x	x	x	x	

^a^ research articles; ^b^ review articles; ^1^ challenges; ^2^ opportunities.

## Data Availability

No new data were generated or analyzed in this study. This article is based solely on a review of the existing scientific literature.

## Supplementary Materials

The following supporting information can be downloaded at: https://www.mdpi.com/article/10.3390/ijerph22091455/s1, Table S1: PRISMA-ScR Checklist.
