# Supplementary material for: Nature-Based Solutions in Workplace Settings: A Scoping Review on Pathways for Integrated Quality, Environmental, Health, and Safety Management
Source: Int J Environ Res Public Health. 2025 Sep 19;22(9):1455. doi: 10.3390/ijerph22091455 (PMC12470130; doi:10.3390/ijerph22091455)
Supplement: Supplementary file 1 [file ijerph-22-01455-s001.zip › ijerph-3787511-supplementary.pdf]

Table S1. PRISMA-ScR Checklist.

Section/Topic	Item	PRISMA-ScR Checklist Item	Reported in Section
Title	1	Identify the report as a scoping review.	Title, Abstract
Abstract	2	Structured summary, indicating scoping review.	Abstract
Introduction	3	Rationale for the review in the context of current knowledge.	Introduction
	4	Explicit statement of objectives/questions.	Introduction, End
Methods	5	Protocol and registration (protocol information and registration details).	Methodology (+ OSF link)
	6	Eligibility criteria for sources of evidence (inclusion/exclusion criteria).	Methodology
	7	Information sources (databases, dates, other sources).	Methodology
	8	Search strategy, including search terms and limits, for at least one database.	Methodology, Table 1
	9	Selection process (screening, eligibility, inclusion).	Methodology
	10	Data charting process (how data were extracted).	Methodology
	11	Data items (variables, outcomes, etc.).	Methodology, Tables
	12	Critical appraisal of sources of evidence (if done).	Methodology
	13	Synthesis of results (how data were handled and summarized).	Methodology, Results
Results	14	Selection of sources (numbers, reasons for exclusion, PRISMA flow).	Methodology, Results, Figure 1
	15	Characteristics of sources of evidence (study details).	Results, Tables
	16	Critical appraisal within sources (if done).	Results, Discussion
	17	Results of individual sources of evidence.	Results, Tables, Discussion
	18	Synthesis of results (summarized, mapped evidence).	Results, Discussion
Discussion	19	Summary of evidence (key themes, implications, limitations).	Discussion, Conclusion
	20	Limitations of the scoping review.	Discussion, Conclusion
	21	Conclusions and potential implications/future directions.	Conclusion
Funding	22	Funding sources and roles.	Funding
